# The Korean Society for Biomaterials joins forces with BioMed Central

**DOI:** 10.1186/2055-7124-18-4

**Published:** 2014-06-13

**Authors:** Insup Noh

**Affiliations:** Department of Chemical and Biomolecular Engineering, Convergence Institute of Biomedical Engineering and Biomaterials, Seoul National University of Science and Technology, 232 Goneung-ro, Nowon-gu Seoul, 139-743 Korea

## Abstract

It is my pleasure to usher in a new era for *Biomaterials Research* in 2014: gold open access. The journal has a long history of publishing research across the broad spectrum of biomedical materials. Supported by the Korea Research Foundation, it was founded in 1996 as the official journal of the Korean Society for Biomaterials. *Biomaterials Research* now joins BioMed Central’s portfolio of open access journals.

*Biomaterials Research* explores both the discovery and development of basic medical materials – a field that is growing rapidly, arising both from its scientific importance and the needs of our aging society. We are certain that this transformation into an open access journal will bring *Biomaterials Research* greater visibility and increase its impact internationally, enabling us to attract new authors and readers from around the world.

All articles are accessible online to readers at no cost, ensuring the journal reaches the widest possible audience. Content will be available not only to scientists, but also to those working in industry and policy makers, facilitating the dissemination of important scientific information to all. Authors retain copyright for their work and grant anyone the right to reproduce and disseminate the article (under a CC-BY licence), provided that the original work is correctly cited.

*Biomaterials Research* welcomes both original research articles and invited reviews in diverse fields of experimental and clinical biomaterials, including polymers, ceramics, metals and their combined composites for medical applications from nano- through to macro-scale. The research topics of our publications cover medical materials science, and technology of biomaterials related to regeneration of diverse tissues such as bone, cartilage, skin and blood vessels. Other important research topics include medical science and technology of medical devices and implants, drug delivery systems, nano-biomaterials, bioimaging, stem cells, 3D bioprinting, interfaces between surfaces and cells, biocompatibility, surface modification, immunological responses of biomaterials, *in vitro* and *in vivo* evaluations of biomaterials, as well as their clinical applications. We will continue to expand the journal’s scope to cover relevant and state-of-the-art topics in biomaterials research as they emerge.

The timely dissemination of research is critical for improving the scientific impact and competitiveness of *Biomaterials Research*. As such, the journal is committed to providing an efficient, high-quality peer-review service. Manuscripts will be processed immediately upon submission. Overseen by an Associate Editor, manuscripts will be evaluated by at least two independent reviewers, experts in the relevant field, who will be asked to appraise the manuscript in a timely manner. The journal’s constructive and informative peer review will help authors improve the scientific quality of the manuscript. The methods and results of the manuscript should be clearly described, and placed in the context of the existing scientific literature. Sufficient experimental and scientific descriptions should be provided to allow readers to evaluate their work with ease, with conclusions appropriately supported by the data presented and the analyses. Acceptance for publication is based upon the scientific content, originality and scientific/clinical impact on the biomaterials field.

This new alliance between the Korean Society for Biomaterials and BioMed Central will strengthen the international reach and recognition of *Biomaterials Research.* By removing access barriers, the journal can be a community hub where interdisciplinary researchers from academia, industry and clinicians can all access, and comment upon, the latest biomaterials research. We welcome your submissions of original research and review manuscripts as we enter this new era in the journal’s history.

Insup Noh, PhD

Editor-in-Chief of *Biomaterials Research*

